# Volatile organic compound detection of Buruli ulcer disease: Headspace analysis of *Mycobacterium ulcerans* and used gauzes of Buruli-compatible ulcers

**DOI:** 10.1371/journal.pntd.0012514

**Published:** 2024-09-23

**Authors:** Stan F. J. Chudy, Delphin M. Phanzu, Arend H. J. Kolk, Ghislain E. Sopoh, Yves T. Barogui, Oren Tzfadia, Miriam Eddyani, Krista Fissette, Bouke C. de Jong, Paul Brinkman

**Affiliations:** 1 Department of Respiratory Medicine, Academic Medical Centre, Amsterdam, The Netherlands; 2 Institut Medical Evangélique de Kimpese (IME), Kimpese, Democratic Republic of Congo; 3 Centre de Recherche en Santé de Kimpese (CRSK), Kimpese, Democratic Republic of Congo; 4 Centre De Dépistage et de Traitement de l’Ulcère de Buruli (CDTUB), Allada, Benin; 5 World Health Organisation (WHO), Brazzaville, Congo; 6 Mycobacteriology Unit, Institute of Tropical Medicine, Antwerp, Belgium; 7 Independent consultant, Antwerp, Belgium; Centre hospitalier de Cayenne, FRANCE

## Abstract

Diagnosing Buruli ulcer (BU) is complicated by limited access to the sensitive IS*2404* qPCR. Experienced clinicians report a distinct odour of Buruli ulcers. We explored the potential of headspace analysis by thermal desorption-gas chromatography-mass spectrometry to detect volatile organic compounds (VOCs) from *Mycobacterium ulcerans* both *in vitro* and clinically.

This study was conducted in two phases: a discovery and validation phase. During the discovery phase, VOCs that enable identification of *M*. *ulcerans* cultures were determined. During the validation phase, these VOCs were evaluated in clinical samples for which we used gauzes from patients with skin ulcerations in the Democratic Republic of Congo.

Seven *M*. *ulcerans* headspace samples were compared with four from sterile growth medium and laboratory environmental air. The univariate analysis resulted in the selection of 24 retained VOC fragments and a perfect differentiation between cultures and controls. Sixteen of 24 fragments were identified, resulting in eleven unique compounds, mainly alkanes. Methylcyclohexane was the best performing compound. Based on these 24 fragments, headspace samples originating from gauzes of 50 open skin lesions (12 qPCR positive and 38 negative) were analysed and an AUC of 0.740 (95%-CI 0.583–0.897) was obtained. As this is an experimental study, future research has to confirm whether the identified compounds can serve as novel biomarkers.

## Author summary

In this study the odour of bacteria that cause Buruli ulcer was examined. First, the air of bacteria cultured in the lab was analysed and compared with controls. Next, air was sampled from gauzes worn by people in the Kongo Central province, the Democratic Republic of Congo. Their lesions were compatible with Buruli ulcer disease but only 12 out of 50 were infected with the Buruli bacterium. In the odour of the Buruli bacteria and in gauzes from people with Buruli ulcer, several chemicals were present more abundantly than in gauzes from people with wounds caused by other diseases. Future research has to determine whether these chemicals can become part of a new test for Buruli ulcer disease.

## Introduction

Buruli ulcer (BU), a necrotising skin disease caused by *Mycobacterium ulcerans*, can result in severe morbidity if not treated at an early stage. It usually starts as a small nodule, developing over weeks to months into an ulcer [1,2]. The disease has been reported in more than 33 countries, mainly in tropical and subtropical regions. In 2023, ten countries in West and Central Africa and parts of Australia were most affected and BU is also endemic in French Guiana, Japan, and Papua New Guinea [1]. Especially on the African continent most endemic areas are remote rural regions with limited healthcare facilities, where multiple factors lead to under-recognition, under-diagnosis and under-reporting of this neglected tropical skin disease (skin NTD) [3–8].

Early detection and adequate care are crucial, as patients who are diagnosed late are at risk to suffer long-term functional disabilities [9,10]. Based on characteristics of the ulcer and the patient–most notably the necrotic base, undermined edges, age <15 years and the absence of pain–a clinical diagnosis by an experienced clinician or expert panel can be made rather reliably [11,12]. Unfortunately, experienced clinicians are scarce in many affected areas and expert panels are not used routinely. In Australia IS*2404* qPCR allows for early diagnosis, even before nodules have ulcerated [13]. The insertion sequence IS*2404* has over 200 copies per bacterial genome and is the target of choice, with excellent sensitivity and specificity, yet requires sophisticated laboratories with strict quality control as it is prone to molecular contamination and false positives. Patients at the highest risk for BU on the African continent–in remote, rural areas–have limited rapid access to quality controlled molecular diagnostics [1]. Detection of acid-fast bacteria to confirm the presence of mycobacteria by Ziehl-Neelsen microscopy has a sensitivity of 60% at best and cannot distinguish between BU and other (atypical) mycobacterial skin ulcerations like cutaneous tuberculosis and *M*. *marinum* infections [2,14]. A bacteriological culture can potentially be diagnostic, yet is difficult to perform, availability is low, it takes 6–12 weeks and is still only positive in less than half of qPCR confirmed patients [2]. A reliable, non-invasive point-of-care (PoC) test would allow for direct screening of BU during active case-finding activities. Although some antigen- and antibody-based tests have been studied, to date none have been developed into point-of-care diagnostics [15,16].

Anecdotal reports of experienced physicians mention a specific BU odour, these have led to including the a ‘characteristic BU odour’ in two recent scientific studies [12,17]. These studies aimed to estimate the predictive value of epidemiological criteria. Next to a typical odour, clinical characteristics like the location of the lesion, whether it was painless, ulcerated or undermined were included. One study compared the odour in relation to a high or low BU-likelihood and reported an adjusted odds ratio of 4.7 (95%CI 1.7–12.9), making it the strongest clinical predictor of Buruli ulcer [17]. The second concluded that among the additional characteristics studied the strongest association with a positive PCR result was the characteristic odour of BU with an odds ratio of 16.4 (95%CI: 7.5–36.6) [12].

An odour is a combination of volatile organic compounds (VOCs). To analyse this mixture of VOCs different approaches can be used. Mass spectrometry (MS) can identify individual compounds, whereas chemical sensor-based electronic nose (eNose) technology can recognise odour profiles. Complex analytical chemistry techniques like gas chromatography-mass spectrometry (GC-MS) fit the aim for the discovery of novel targets and mechanistic pathways, whereas quick, easy to operate, and low-cost eNose devices are more suitable for assessment of disease states at point-of-care [18].

Specific VOCs have been identified in the headspace of mycobacterial cultures [19–26], yet to date *M*. *ulcerans* was not studied. We hypothesised that *M*. *ulcerans* produces specific VOCs, which can be identified by GC-MS-based headspace analysis of *M*. *ulcerans* cultures and clinical samples (used gauzes). Identification of these VOCs could be a first step in the development of a new diagnostic tool for BU.

## Methods

### Ethics statement

The collection of clinical samples was embedded in an observational study on the epidemiology of BU in the Kongo Central province. This study (Protocol “Approche micro-épidémiologique pour comprendre la dynamique de transmission de l’ulcère de Buruli dans la province du Kongo Central en RDC” [A micro-epidemiological approach to understand the transmission dynamics of Buruli ulcer in the province of Kongo Central in the DRC]) was reviewed and approved by the ethics committee of the School of Public Health at the University of Kinshasa, with reference ESP/CE/089/2015. The institutional review board of the Institute of Tropical Medicine in Antwerp confirmed the decision on this protocol, reference number 929/14. Written information was provided to all participants and a health professional provided information about the study verbally in either Kikongo or Lingala. All participants gave informed consent by providing a signature or fingerprint. For children < 18, written consent was obtained from a legal guardian.

### Sample collection

In the discovery phase, the headspace of *M*. *ulcerans* cultures was analysed. In the subsequent validation phase, the results obtained from the cultures were evaluated in clinical samples, using gauzes. To provide more clarity: a gauze is the fluid-absorbing (sterile) fabric in direct contact with a lesions while a bandage is the piece of cloth wrapped around the affected limb to fixate the gauzes and protect the lesion.

### Cultures—Discovery phase

Cultures from *M*. *ulcerans* CT2007-03151, stored at the Institute of Tropical Medicine (ITM) in Antwerp, were prepared in 250 and 500 mL flasks (Duran GL45, Wertheim/Main, Germany) in 100 mL of Middlebrook 7H9 broth with OADC (7H9+OADC) and incubated at 30°C (range 28–32°C) for three to seven weeks before testing. Two 500 mL flasks each containing sterile 100 mL 7H9+OADC of six to seven weeks old were used as controls, as were samples from laboratory air.

**Fig 1:**
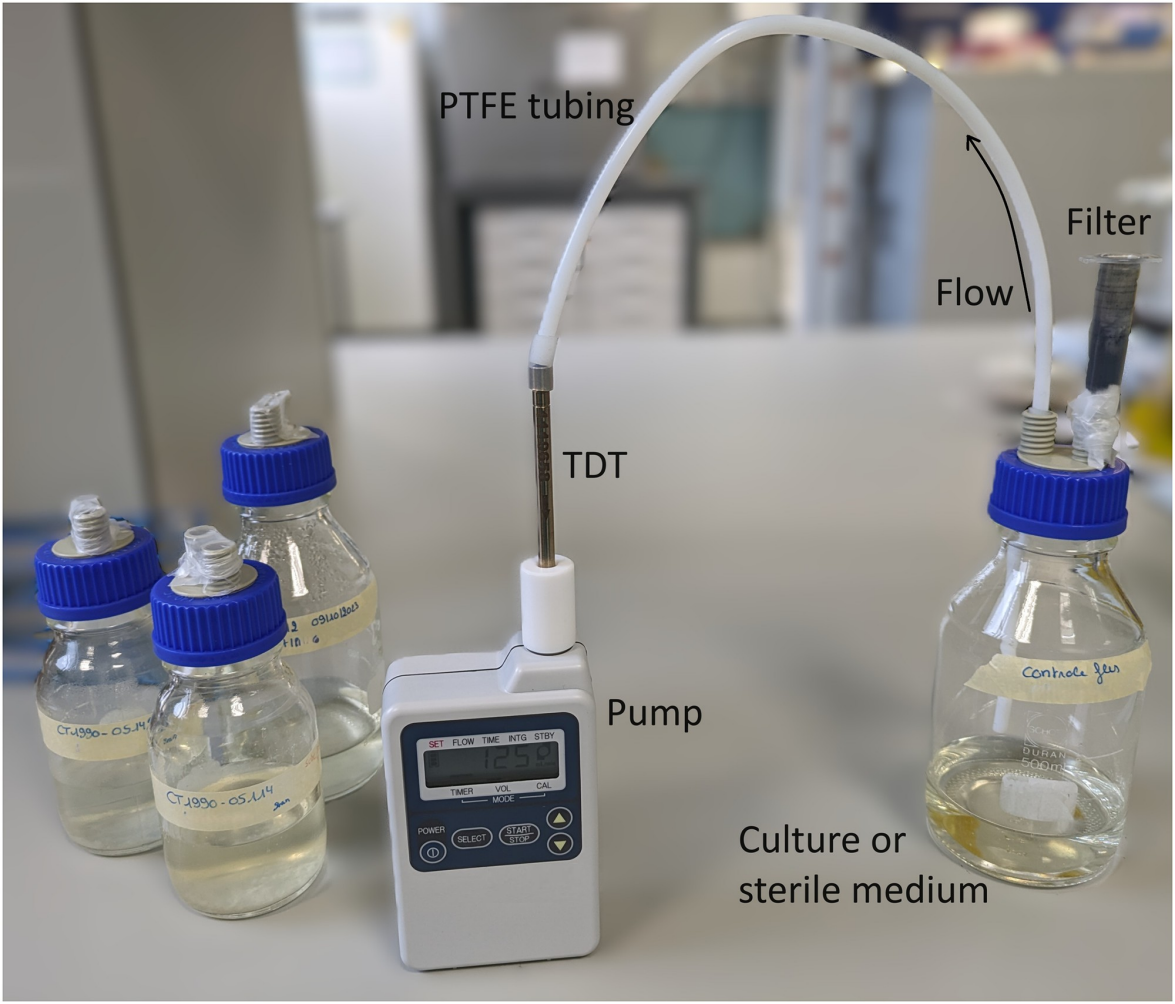
Set-up for headspace sampling of M. ulcerans cultures. For the sampling of the headspace of the gauzes (clinical samples), the same materials were used, but instead of a bacterial culture, a used gauze was put in the bottle. TDT = thermal desorption tube. Filter = carbon filter to clean inflow of air.

To collect the headspace of the cultures and control samples, polytetrafluoroethylene (PTFE) tubing connected the culture flasks sealed with a double outlet cap (Duran GL45 PP 2 ports GL14, Wertheim/Main, Germany) to a thermal desorption tube (Camsco, ¼”x3.5” Stainless Steel Thermal Desorption (TD) Tube, Carbograph 40/60 & Carbopack 40/60) and a volumetric air pump (GSP-300FT-2, GASTEC corporation, Kanagawa, Japan). With a flow rate of 125 mL/min and a sampling time of 2 minutes, a volume of 250 mL of headspace was collected from each of the samples (Fig 1).

### Clinical samples—Validation phase

For the validation phase the headspace sampling method was adapted to capture VOCs from used gauzes. This part of the study was conducted in the districts surrounding the IME Kimpese hospital, Kongo Central province, the Democratic Republic of Congo (DRC).

Patients with non-traumatic skin ulcerations, including presumptive BU lesions, undergoing changing of gauzes and/or surgery as part of their regular care were enrolled. No age restriction was applied. Patients with non-ulcerative lesions (intact nodule, plaque, oedema), although sometimes presumptive-BU lesions, were excluded. Patients with visible (remnants of) traditional or herbal treatment in the ulceration were excluded, as these products could influence the wound odour.

The majority of patients (31 of 50) was recruited in peripheral health centres in the districts surrounding the IME Kimpese hospital, others in one of the two hospitals in the region. Field visits were conducted by the sampler and local health care professionals trained on the diagnosis and management of BU. Samples were taken between 7th September and 20th December 2017.

We used the same laboratory materials mentioned before to analyse the headspace of used gauzes. These gauzes were transferred into glass laboratory bottles to isolate the sample and prevent contamination with environmental VOCs where they were stored for 30–120 minutes after which the closed lid was carefully exchanged for the screw cap with two ports. A carbon filter was connected to one port to filter incoming air and prevent contamination. Identical to the discovery phase, the outlet was connected to a thermal desorption tube and the volumetric air pump. The flow rate was set at 125 mL/min and the sampling time was 2 minutes, resulting in a sampling volume of 250 mL. Polytetrafluoroethylene (PTFE) tubing was chosen for its low binding capacity of organic molecules and heat resistance to prevent contamination and allow for proper sterilisation together with the glass bottles and screw caps. After each use, bottles and PTFE tubing were rinsed with water and soap before being sterilised in a steam autoclave at 120°C to prevent contamination of subsequent samples.

### Diagnosis of Buruli Ulcer

After removing the gauzes from the wound, diagnosis of BU was made using Copan Floq swabs sent in ethanol for IS*2404* qPCR at ITM, as previously described [27].

### Gas chromatography-mass spectrometry analysis

As previously described by Fenn et al. [28] following VOC capture, all samples were first desorbed using the Markes TD100 autosampler and desorber (Cincinnati, Ohio, USA) before GC-MS analysis. Thermal desorption tubes were heated to 250°C for 5 minutes with a flow of 30 mL/min. VOCs were captured on a cold trap at 25°C and re-injected by rapidly heating the trap to 280°C for three minutes. VOCs were injected splitless through a transfer line at 120°C onto an Inertcap 5MS/Sil GC column [30 m, ID 0.25 mm, film thickness 1 μm, 1,4-bis(dimethylsiloxy)phenylene dimethyl polysiloxane (Restek, Breda, The Netherlands)] with a flow of 1.2 mL/min. Oven temperature was kept isothermal at 40°C for 5 minutes, then increased to 280°C at 10°C/min and kept isothermal at 280°C for 5 minutes. Molecules were ionised using electron ionisation (70 eV), and the fragment ions were detected using a quadrupole mass–spectrometer (GCMS–GP2010, Shimadzu, Den Bosch, the Netherlands) with a scan range of 37–300 Da.

Compounds of interest were identified using the GC-MS solutions (Shimadzu, Den Bosch, the Netherlands) platform as previously described [29] and compared to analytical standard where possible. Any compounds that had match score < 80 or could not be accurately detected due to co-elution were excluded from further analysis.

### Statistical analysis

Statistical analysis was performed through the R studio interface using R (version 3.6.1). Raw GC-MS spectra were processed using the R “XCMS’ package (Scripps Center for Metabolomics, La Jolla, CA, USA) as previously reported [29], and underwent denoising, peak detection and alignment to create a three-dimensional data matrix containing sample metadata, retention time (rt) and mass-to-charge ratio (m/z), ahead of downstream analysis. Visual inspection of the chromatograms following the “XCMS” pipeline, allowed for the exclusion of any failed or technical errors that might have occurred.

**Fig 2:**
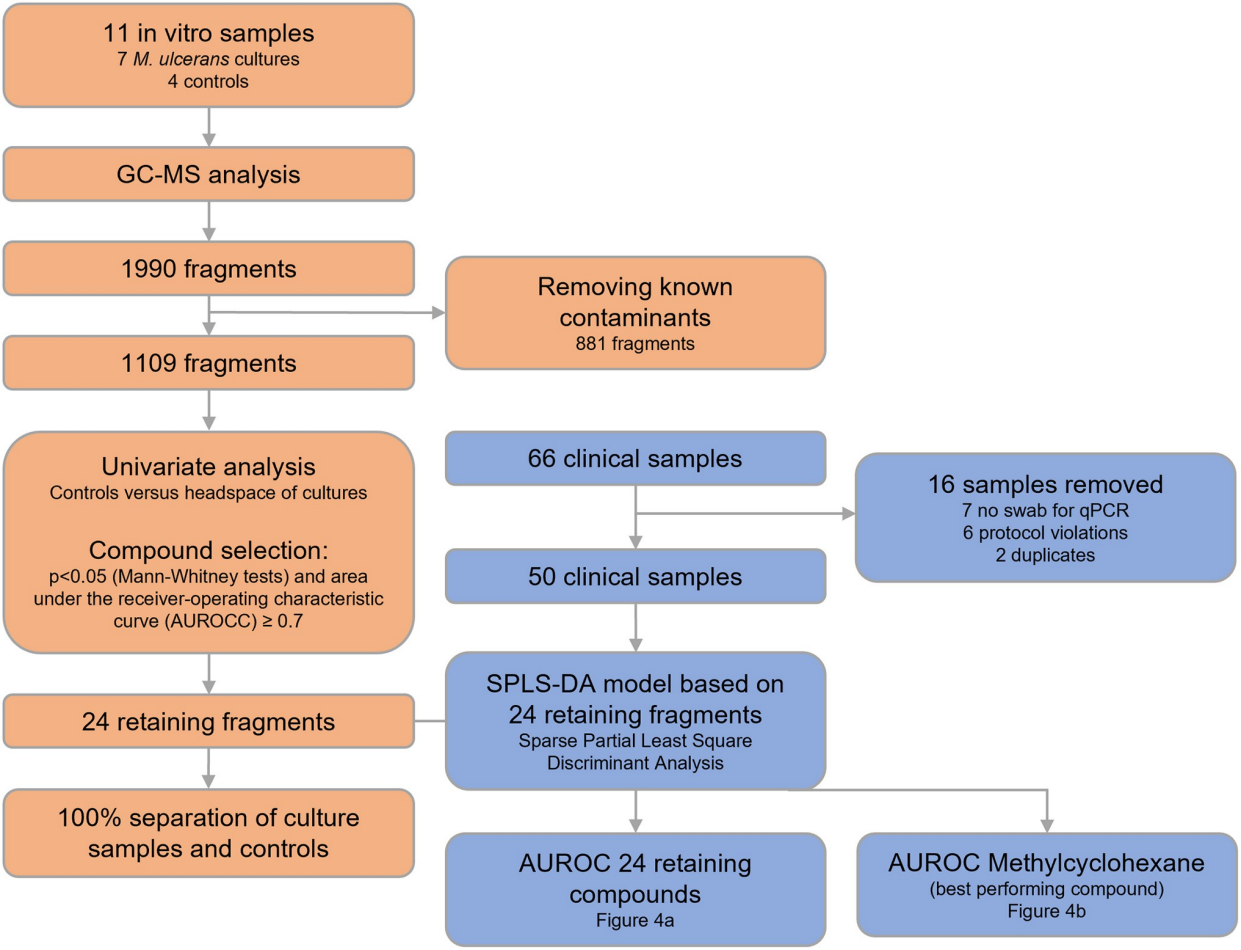
Flowchart of data analyses. Orange: discovery phase (in vitro, M. ulcerans cultures). Blue: validation phase (clinical, gauzes).

The GC-MS analysis initially returned 1990 fragments. After removal of known contaminants, 1109 fragments were left and the gauze and *in vitro* GC-MS results were split. Next, univariate analysis on *in vitro* GC-MS fragments was performed with the headspace of *M*. *ulcerans* culture versus controls (laboratory air and sterile growth medium). GC-MS fragments showing a p<0.05 (Mann-Whitney tests) and area under the receiver-operating characteristic (AUROC) curve ≥ 0.7 [30], were then subjected to Sparse Partial Least Square Discriminant Analysis (SPLS-DA). The derived *in vitro* based SPLS-DA model was subsequently validated on the gauze samples, whereby ROC modelling was applied again as performance test. Finally, the individual GC-MS fragments driving the SPLS-DA model were examined through box-and-whisker plots, for both the *in vitro* and gauze samples (see Fig 2).

## Results

### Cultures—Discovery phase

**Fig 3:**
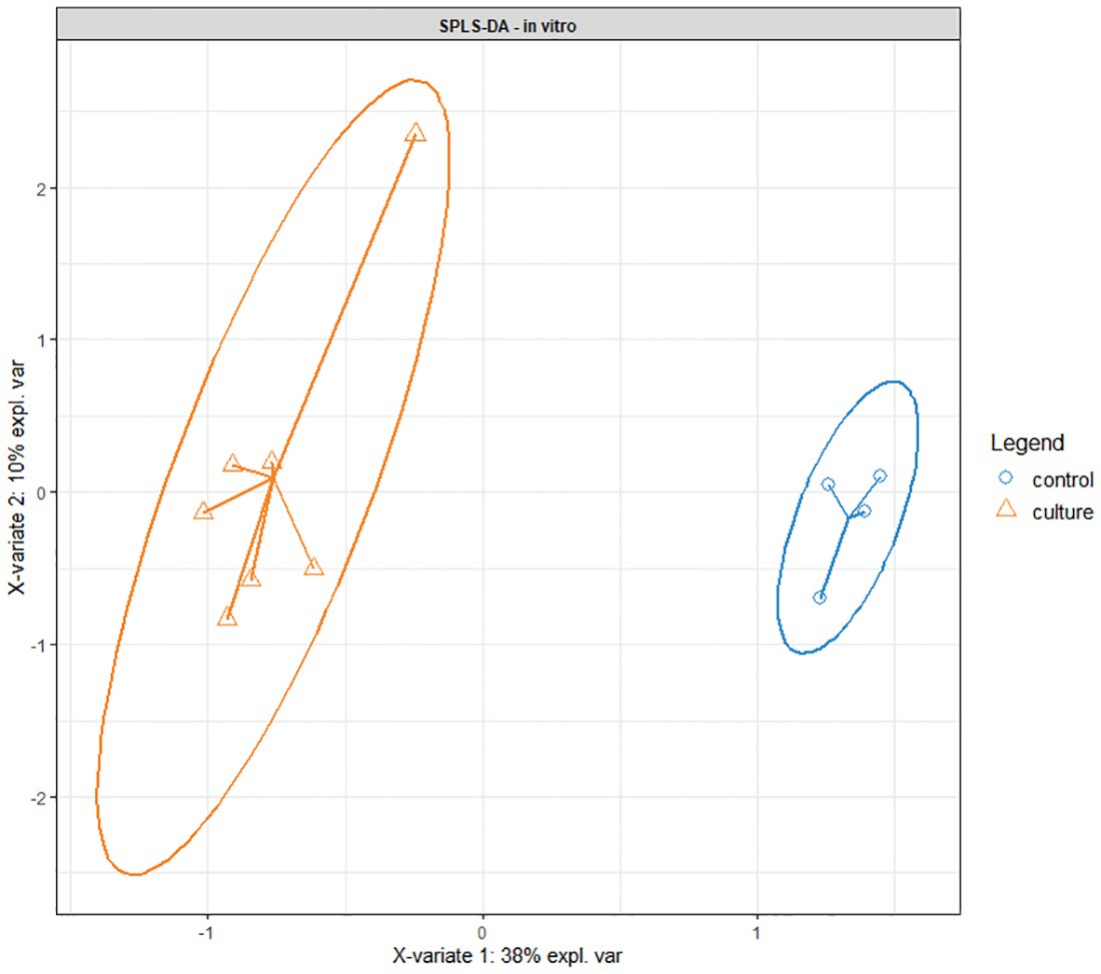
SPLS-DA plot of in vitro samples based on 24 fragments that were retained after univariate analysis.

Seven *M*. *ulcerans* headspace samples were compared to four from sterile growth medium and laboratory air. A univariate analysis was performed on the 1109 GC-MS fragments with predefined criteria: p<0.05, AUROCC ≥ 0.7, and upregulation in the bacterial cultures. This resulted in 24 retained fragments. SPLS-DA driven multivariate analysis showed a perfect differentiation of the two sample types (cultures versus controls, Fig 3).

These 24 fragments were used as the basis for the analysis of the gauze samples and identified using GCMS Postrun Analysis software (Shimadzu) with the NIST library as reference (NIST23, version 3.0). Sixteen fragments met the predefined criteria: a similarity of ≥95% and an identification in ≥2 samples. Multiple fragments were identified as belonging to the same compound, in total eleven unique compounds were found (Table 1).

**Table 1:** Putatively identified compounds that discriminate between *M*. *ulcerans* cultures and controls with the similarity of their GC-MS profiles compared to the NIST library, their P-values, literature references of previous detection in the volatilome of mycobacterial cultures [19, 23, 31–33] and other microorganisms according to the mVOC 3.0 Database [34] and human skin [35], highlighting the six most common bacterial co-infections of Buruli ulcer lesions [36–39].

CAS	Compound	Similarity	P-value	Emitted by (not exhaustive): Mycobacteria	Other bacteria or skin	SA	PA	BHS	KP	EC	PM
123-51-3	3-methyl-1-butanol	96%	0.023	MB [19] MI, MM, MD, MS [23]	-	**yes**	**yes**	no	**yes**	**yes**	no
108-87-2	Methylcyclohexane*	97%	0.006	*No references found*	HI, PL, MC	no	no	no	no	no	no
591-47-9	4-methylcyclohexene	96%	0.006	*No references found*	-	no	no	no	no	no	no
96-37-7	Methylcyclopentane	98%	0.045	MAP, MI, MT, MM, MD, MS [23]	-	no	no	no	no	no	no
1638-26-2	1,1-dimethylcyclopentane	95%	0.042	*No references found*	-	no	no	no	no	no	no
2453-00-1	1,3-dimethylcyclopentane	95%	0.023	*No references found*	-	no	no	no	no	no	no
624-92-0	Dimethyl disulfide	98%	0.006	MD, MP [23], MAP [31] MAU, MN [32], MAV [33]	Many microorganisms	no	**yes**	**yes**	no	no	no
142-82-5	Heptane	99%	0.006	MAP [23, 31] MB, MI, MT, MM, MD, MF, MS [23]	HI, PL, MC, human skin	no	no	no	no	no	no
589-43-5	2,4-dimethylhexane	97%	0.006	MAP [31]	Human skin	no	no	no	no	no	no
111-84-2	Nonane	95%	0.006	*No references found*	Human skin	no	no	no	no	no	no
108-88-3	Toluene	97%	0.006	*No references found*	Many microorganisms, human skin	no	no	no	no	no	no

BHS = beta-hemolytic streptococcus, EC = *Escherichia coli*, HI = *Haemophilus influenzae*, KP = *Klebsiella pneumoniae*, LP = *Legionella pneumoniae*, MAV = *M*. *avium*, MAP = *M*. *avium ssp*. *Paratuberculosis*, MAU = *M*. *aurum*, MB = *M*. *bovis BCG*, MC = *Moraxella catarrhalis*, MD = *M*. *diernhoferi*, MF = *M*. *fortuitum*, MI = *M*. *intracellulare*, MM = *M*. *marinum*, MN = *M*. *neoaurum*, MS = *M*. *smegmatis*, MT = *M*. *terrae*. SA = *Staphylococcus aureus*, PA = *Pseudomonas aeruginosa*, PM = *Proteus mirabilis*.

* = strongest discriminating compound

### Clinical samples—Validation phase

We collected 66 headspace samples, 16 were excluded (7 had no corresponding qPCR result, 6 due to protocol violations and 2 duplicates), leaving 50 headspace samples from used gauzes of 48 patients for further analyses (two patients had two lesions each, all four were qPCR negative). Twelve samples came from ulcers for which the concurrent swab tested qPCR positive, and 38 negative. The clinical diagnosis compared to the qPCR result had a sensitivity of 42% with a specificity of 87%. Median age, gender ratio, and self-reported disease length were similar for both groups (Table 2). Also, the large majority of lesions was located on the lower limbs. Most patients in both groups had already received some form of treatment from a health centre and 25% and 39% of patients in the qPCR positive and negative groups respectively had previously consulted a traditional healer. Six patients received BU-specific antibiotic treatment; four patients in the qPCR confirmed group received respectively 1, 4 and twice 8 weeks of treatment and two patients in the qPCR negative group received 1 and 8 weeks of treatment. This is a small minority because most of the sampling was performed prospectively during active case finding.

**Table 2:** Patient characteristics by concurrent *IS2404* qPCR result.

	qPCR POS (n = 12)		qPCR NEG (n = 38)		All (n = 50)	
	n	%	n	%	n	%
Sex, female: male (% female)	5: 7	42	19: 19	50	24: 26	48
Age, median years (range)	29	(10–72)	32	(3–81)	32	(3–81)
Symptom duration, median days (range)	135	(4–672)	90	(11–6076)	90	(4–6076)
Lesion						
Lower extremities	10	83	33	87	43	86
Upper extremities	1	8	4	11	5	10
Other	1	8	1	2	2	4
Ulceration	11	92	38	100	49	98
Previously received care						
Health centre	1	92	30	79	41	82
Traditional medicine	3	25	15	39	18	36
Started on BU treatment	4	33	2	5	6	12
Clinical diagnosis						
BU	8	67	11	29	19	38
Non-BU	4	33	2	71	31	62

**Fig 4:**
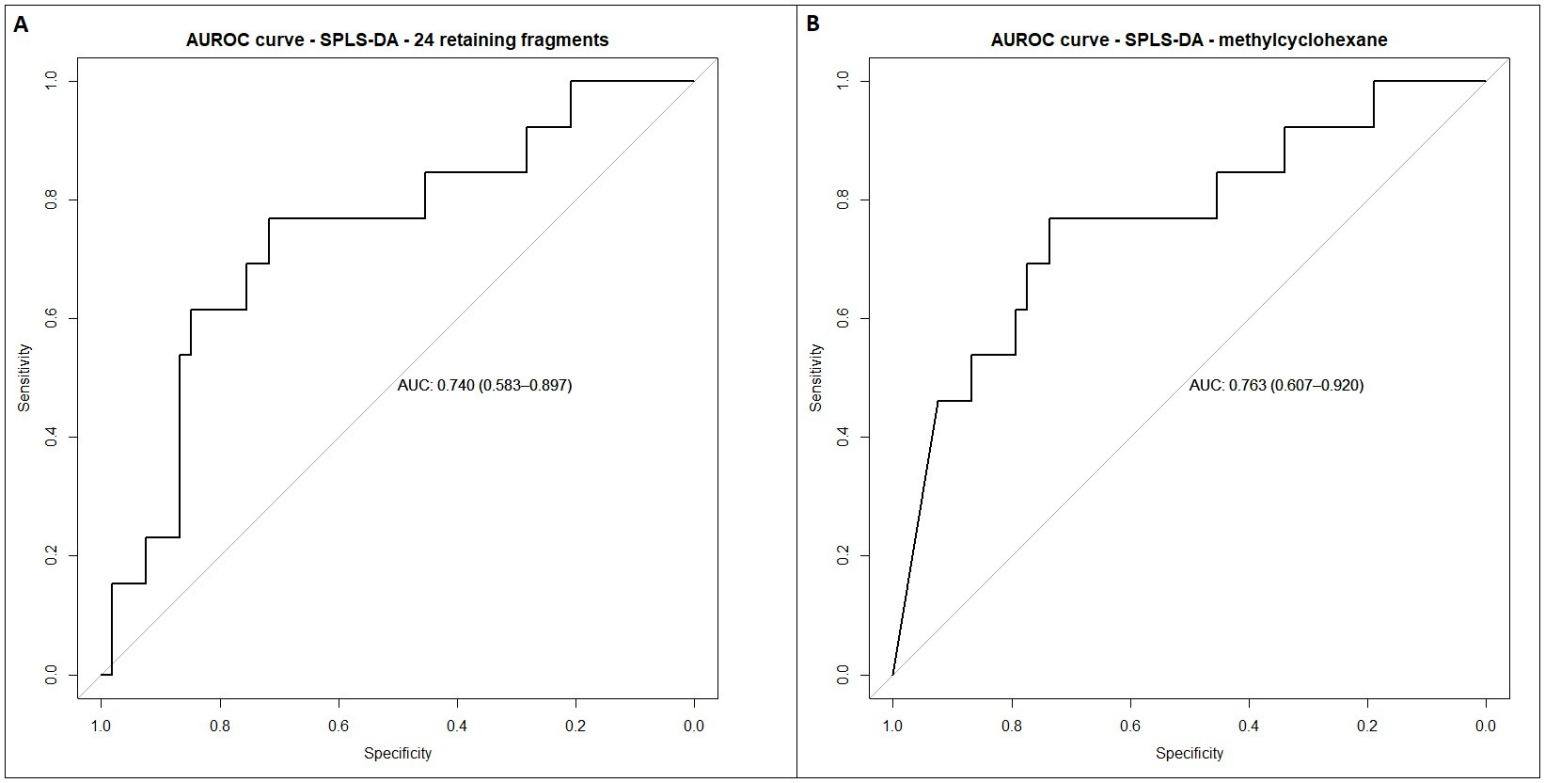
a. AUROC curve of SPLS-DA analysis with 24 retained compounds. b. AUROC curve of SPLS-DA analysis with best performing compound, methylcyclohexane.

Using the retaining 24 ion fragments as input for multivariate SPLS-DA (mixOmics 6.14.1; functions: tune.splsda + splsda [40]) one SPLS-DA component was derived and resulted in a significant p-value of p<0.01 and an AUROCC of 0.92 (95% CI: 0.77–1.00). The SPLS-DA analysis on the gauze samples yielded a differentiation with an AUC of 0.740 (95% CI: 0.583–0.897, Fig 4A).

**Fig 5:**
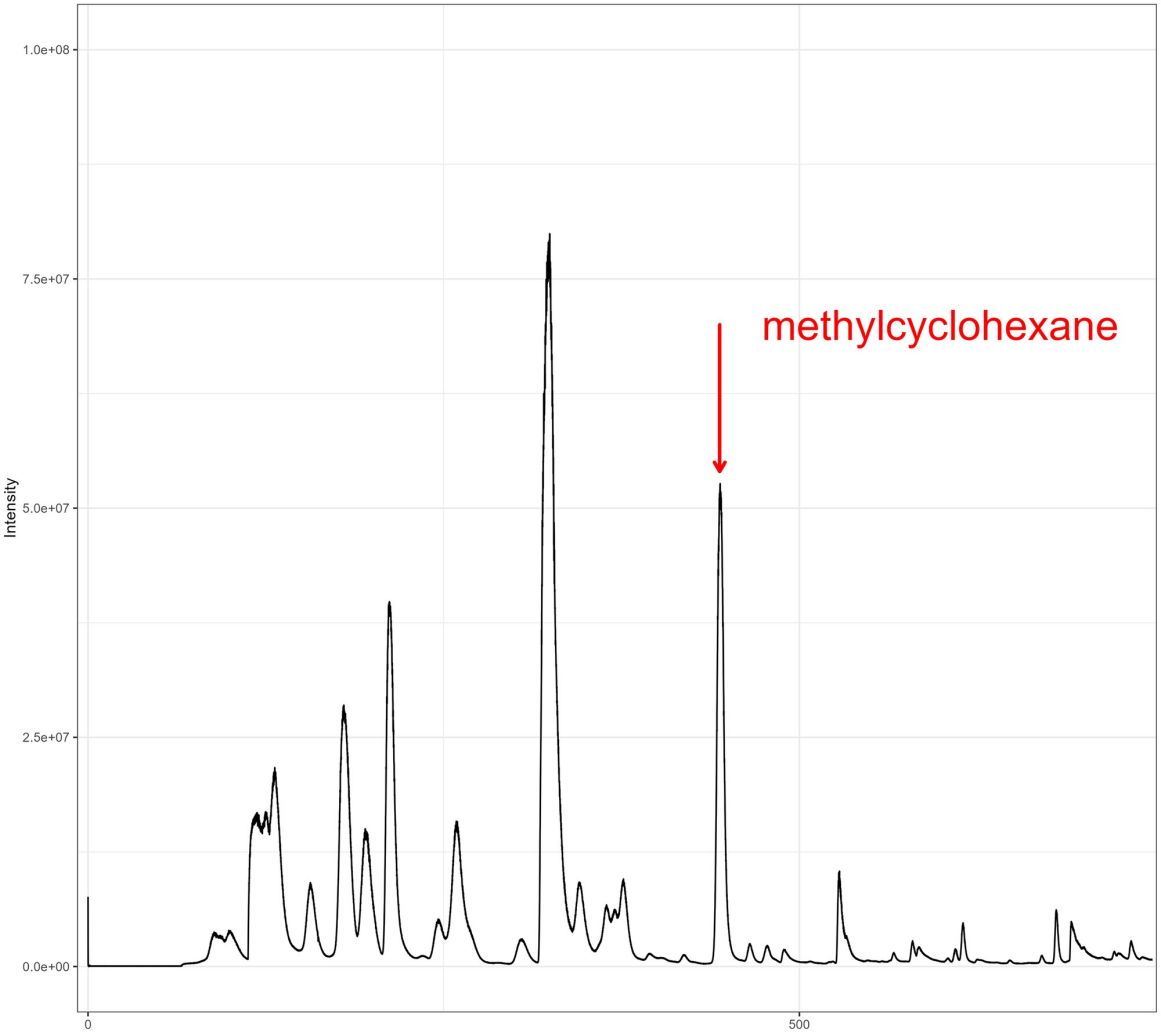
Excerpt from a chromatogram of M. ulcerans culture headspace, indicating the fragment at 444 seconds that was identified as methylcyclohexane.

Among the 24 compounds, the best performing compound from the *in vitro* analysis yielded a slightly better result with an area under the curve (AUC) of 0.763 (95% CI 0.607–0.920, Fig 4B). This fragment was identified as methylcyclohexane. To illustrate the GC-MS output of a sample containing methylcyclohexane, a chromatogram is shown (Fig 5).

## Discussion

By using thermal desorption tube gas chromatography–mass spectrometry (TD-GC-MS) driven headspace analysis we identified 24 fragments that best discriminated between *M*. *ulcerans* cultures and controls; and between qPCR-positive and -negative BU-compatible wounds (AUC of 0.740 (95% CI: 0.583–0.897)). From these 24 fragments, eleven unique compounds were identified: alkanes, cycloalkanes, an alcohol, a disulfide and an aromatic (Table 1) and the best performing compound was methylcyclohexane. *In vitro*, methylcyclohexane enabled a perfect discrimination between *M*. *ulcerans* cultures versus media and laboratory air (100% accuracy). Applying headspace analysis on gauzes, this compound also distinguished between qPCR-confirmed Buruli ulcer disease and other ulcers with an AUC of 0.763 (95%-CI 0.607–0.920). These outcomes merit further analyses of VOCs originating from skin lesions, potentially for the development of a non-invasive biomarker screening tool for BU.

To the best of our knowledge, this is the first VOC study on *M*. *ulcerans*. The technique applied to detect VOCs during this study, TD-GC-MS testing, has been used for (bio)medical research before. VOCs from breath [20,24,41,42] and skin [43,44] of patients with tuberculosis, the most common mycobacterial infection in humans, has yielded promising results, as have other studies on pulmonary diseases like asthma, chronic obstructive pulmonary disease, and infectious diseases [45]. Dermatological research on VOCs has focussed on sampling healthy skin [35], as well as emissions from pathological skin bacteria and infected wounds [46–48], precursors of pressure ulcers [49, 50] and skin cancers [51,52]. The present study takes an additional step by conducting an *in vitro* (discovery) and clinical (validation) phase.

In our study, 24 fragments provided a fair discrimination between lesions with a positive versus a negative qPCR result, with methylcyclohexane as the best performing compound. This methylated cycloalkane has not been found previously in relation to *M*. *ulcerans* or other mycobacteria. Dimethyl disulphide, hexane and methylcyclopentane have been identified in other mycobacterial cultures, among which are *M*. *marinum*, *M*. *bovis* and *M*. *tuberculosis* [23]. Also, cyclohexane and several methylated cyclohexanes have been identified in *M*. *tuberculosis* cultures and exhaled breath from patients with pulmonary tuberculosis [19,20,41]. Three of these cyclohexanes and toluene have recently been described in two papers investigating skin emissions in patients with tuberculosis [43, 44].

Using the mVOC library [34] (Table 1), we found that methylcyclohexane and heptane have been identified in cultures of other human pathogens: *H*. *influenzae*, *L*. *pneumoniae* and *M*. *catarrhalis* [53]. These pathogens mainly cause respiratory infections and have not been found as co-infections of BU lesions [36–39]. The latter is also true for microorganisms emitting nonane, this compound is produced by a range of soil bacteria. The analysed gauzes contacted the wound and were always covered by bandages, so contamination by soil bacteria is unlikely. Dimethyl disulfide and toluene are compounds detected in many different microorganisms while the other cyclical compounds were not found in the database. We also searched the volatilome of the most common bacterial co-infections of Buruli ulcer lesions [36–39]. We found 1-butanol, 3-methyl to be present in four out of six volatilomes and dimethyl disulfide in two out of six. None of the cyclical compounds (*n* = 5) nor heptane, hexane, 2,4-dimethyl, nonane or toluene were found in these volatilomes. In samples of healthy human skin [35], heptane, 2,4-dimethylhexane, nonane and toluene have been found but again, none of the cyclical alkanes.

In conclusion, the cyclical alkanes seem to be unique compounds found in *M*. *ulcerans* cultures and used gauzes of BU lesions while the other compounds might be less suitable as potential biomarkers due to lack of specificity.

The existence of a specific ‘Buruli odour’ has been mentioned anecdotally by experienced health care workers in West and Central African countries for years. Noting a typical odour was first used in the ‘Buruli score’, a multivariate prediction model using characteristics of the wound and the patient to diagnose BU as suggested by Mueller et al, 2016 [17]. In the proposed model, the odour is the strongest clinical predictor of high BU likelihood, with an adjusted odds ratio of 4.7 (95% CI 1.7–12.9) [17]. In this paper Cameroonian clinicians describe it as a strong odour, like the smell of rotten fish, cassava or cheese, mixed with smell of pyocyanic bacteria. A second study in Benin found that a characteristic BU odour had the strongest association with a positive qPCR result with an odds ratio of 16.4 (95%CI: 7.5–36.6) [12]. The researchers included a short survey on the BU odour among health care workers and their descriptions varied between disagreeable, nauseating, strongly penetrating and rotten. During our study, the experienced health professionals in the DRC (*n* = 5) nor the field sampler confirmed either of the descriptions above. The smell of pure methylcyclohexane is rather described as faint, benzene-like, 3-methyl-1-butanol smells of alcohol and methylcyclopentane, nonane and heptane smell of gasoline or petroleum, while toluene has a distinctive aromatic odour and dimethyl disulphide smells like garlic [54]. An odour is often complex and consists of a multitude of VOCs, our findings cannot confirm whether or not the smell of the individual compounds are part of the specific, more complex ‘Buruli odour’.

Our study showcases the validation of findings from the *M*. *ulcerans* cultures to clinical measurements of gauzes, which yielded a strong discrimination. This strengthens the plausibility that the identified compounds are indeed a product of *M*. *ulcerans* and not a confounding molecule of the host, the environment or originating from co-infection. Furthermore, analysing gauzes while sealing them off from the environment provided a stable, non-contaminated air flow while avoiding direct contact of hardware with skin and the ulcers. Finally, our set-up with glass jars did not create extra waste, as all materials were reusable and the gauzes were applied as part of the standard treatment.

We realise this study has several limitations. First, non-ulcerative lesions like intact nodules, plaques and oedema were excluded although these can be clinical presentations of BU. BU often starts as an intact nodule, this currently limits the applicability of our findings to the later and more severe stages of the disease. Also, challenges in the field made it difficult to perform the study fully according to protocol. Therefore, nine percent of clinical samples (*n* = 16) were removed because of protocol violations (see Fig 2). Furthermore, differences in environmental settings (outdoor versus indoor, hospital versus out-patient clinics), might have introduced systematic noise. It is assumed that the targeted TD-GC-MS approach and the discovery phase of the study filtered out this noise by focussing solely on VOCs also present *in vitro*. The follow-up and microbiological testing was limited to *M*. *ulcerans* specific qPCR, this decreases the certainty of the clinical diagnosis, mainly the differentiating of patients with non-BU compatible wounds. The specificity is lower than in other studies [11,12], as there was no expert panel involved but only one or two health care workers to make the diagnosis, often on the spot. Another limitation is the imperfect reference standard. While IS*2404* qPCR is the most sensitive test available, in a diagnostic study using evaluation of all diagnostic findings by an expert panel, its sensitivity was only 65% (95% CI 56–73%) [11]. Moreover, six patients had already started BU-specific treatment. We therefore expect some misclassification by qPCR, with some qPCR negative patients likely having had BU.

Many different alkanes and cycloalkanes in breath are known indicators of lipid peroxidation initiated by oxidative stress [55, 56]. Multiple studies have shown (methylated) alkanes in the breath of patients with various diseases including pulmonary tuberculosis [20,42]. Secondly, the identified compounds are also known products of lipid metabolism. Our hypothesis is that most of the compounds we found, including methylcyclohexane, are either products of M. ulcerans’ lipid peroxidation and/or part of the host response to an increase in oxidative stress caused by the *M*. *ulcerans* infection. *M*. *tuberculosis* and *M*. *marinum* are found to (preferentially) consume fatty acids [57,58], we assume this is similar for *M*. *ulcerans*. *In vitro* the substrate for lipid metabolism can be oleic acid (ingredient of the Middlebrook 7H9 medium), *in vivo* cholesterol and fatty acids are most probably the carbon source. Although we found several metabolites in the headspace of *M*. *ulcerans* and in used gauzes of BU lesions, it is unclear how specific this set of compounds is. More work has to be done to compare and verify our findings. Also, sampling the headspace of a modified *M*. *ulcerans* strain with a gene knock-out to impair the fatty acid metabolism could provide more information on whether the identified compounds are produced via a specific metabolic pathway. Gene knock-out research has been published on other mycobacteria, mainly *M*. *tuberculosis* [59,60].

As this is an explorative study, further work has to be done to confirm our findings in other *M*. *ulcerans* strains to minimise the possibility of a co-incidental find or contamination. Not all identified compounds are unique for *M*. *ulcerans*, some are shared by other mycobacteria and others are found in the headspace of healthy human skin or co-infecting bacteria in BU lesions (Table 1). More elaborate controls–like a sample from a contralateral healthy limb–could help to shed a light on the specificity. Also, the absence of methylcyclohexane and other compounds should ideally be verified in cultures of the most common co-infections, infections within the differential diagnosis [1] and other mycobacteria rarely contaminating or causing chronic wounds, like *M*. *tuberculosis* [61] or *M*. *marinum* [62]. Once a more specific (set of) compound(s) has been found, an approach towards a PoC test can be considered. These include, but are not limited to, an e-nose tuned to BU, compact gas chromatography or a colorimetric test reacting to one or more of these biomarkers. An inexpensive and readily available PoC test could reduce the number of expensive qPCR tests and provide a much quicker and more reliable result than conventional laboratory tests. Integrated control and management of skin NTDs, which is key in the strategy of the World Health Organization for the current decade [63]. Therefore, the scope of our novel approach for BU should not be limited to BU alone. Our methodology can be applied to other (neglected tropical) skin diseases for biomarker discovery, potentially leading to a single device for multiple dermatological pathologies.

In conclusion, we identified a set of compounds in the headspace of *M*. *ulcerans* cultures and of gauzes from qPCR-confirmed BU ulcers. These findings need to be confirmed in a follow-up study, both *in vitro* and in clinical studies. Our unique approach can also be used for other (neglected) skin diseases, aiding novel biomarker detection of ulcerative lesions.
